# Supplementary material for: Stacking Model‐Based Classifiers for Dealing With Multiple Sets of Noisy Labels
Source: Biom J. 2025 Mar 12;67(2):e70042. doi: 10.1002/bimj.70042 (PMC11898607; doi:10.1002/bimj.70042)
Supplement: Supplementary file 1 — Supporting Information [file BIMJ-67-e70042-s001.zip › density-based-ensemble-model-main/competitors_real_data/netlab-master/netlab-master/help/metrop.htm]

Netlab Reference Manual metrop


# metrop

## Purpose

Markov Chain Monte Carlo sampling with Metropolis algorithm.

## Synopsis

```
samples = metrop(f, x, options)
samples = metrop(f, x, options, [], P1, P2, ...)
[samples, energies, diagn] = metrop(f, x, options)
s = metrop('state')
metrop('state', s)
```

## Description

`samples = metrop(f, x, options)` uses
the Metropolis algorithm to sample from the distribution
`p ~ exp(-f)`, where `f` is the first argument to `metrop`.
The Markov chain starts at the point `x` and each
candidate state is picked from a Gaussian proposal distribution and
accepted or rejected according to the Metropolis criterion.

`samples = metrop(f, x, options, [], p1, p2, ...)` allows
additional arguments to be passed to `f()`. The fourth argument is
ignored, but is included for compatibility with `hmc` and the
optimisers.

`[samples, energies, diagn] = metrop(f, x, options)` also returns
a log of the energy values (i.e. negative log probabilities) for the
samples in `energies` and `diagn`, a structure containing
diagnostic information (position and
acceptance threshold) for each step of the chain in `diagn.pos` and
`diagn.acc` respectively. All candidate states (including rejected
ones) are stored in `diagn.pos`.

`s = metrop('state')` returns a state structure that contains the
state of the two random number generators `rand` and `randn`.
These are contained in fields
`randstate`,
`randnstate`.

`metrop('state', s)` resets the state to `s`. If `s` is an integer,
then it is passed to `rand` and `randn`.
If `s` is a structure returned by `metrop('state')` then
it resets the generator to exactly the same state.

The optional parameters in the `options` vector have the following
interpretations.

`options(1)` is set to 1 to display the energy values and rejection
threshold at each step of the Markov chain. If the value is 2, then the
position vectors at each step are also displayed.

`options(14)` is the number of samples retained from the Markov chain;
default 100.

`options(15)` is the number of samples omitted from the start of the
chain; default 0.

`options(18)` is the variance of the proposal distribution; default 1.

## Examples

The following code fragment samples from the posterior distribution of
weights for a neural network.

```
w = mlppak(net);
[samples, energies] = metrop('neterr', w, options, 'netgrad', net, x, t);
```

## Algorithm

The algorithm follows the procedure outlined in Radford Neal's technical
report CRG-TR-93-1 from the University of Toronto.

## See Also

`hmc`

---

**Pages:**
Index


---

Copyright (c) Ian T Nabney (1996-9)
